# *In-vivo* and *In-vitro* Antioxidant Activity of Troxerutin on Nickel Induced Toxicity in Experimental Rats

**DOI:** 10.22037/IJPR.2017.2196

**Published:** 2020

**Authors:** Perumal Elangovan, Abdulkadhar Mohamed Jalaludeen, Ramalingam Ramakrishnan, Kasinathan Amutha, Leelavinothan Pari

**Affiliations:** a *Department of Biochemistry and Biotechnology, Faculty of Science, Annamalai University, Annamalainagar – 608002, Tamil Nadu, India. *; b *Department of Biochemistry, St. Joseph’s College of Arts & Science (Autonomous), Cuddalore 607001, Tamil Nadu, India. *; c *Department of Biochemistry, Sri Sankara Arts & Science College, Enathur, Kancheepuram,Tamil Nadu, India.*

**Keywords:** Troxerutin, Antioxidants, Nickel, Free radical, Hydroxyl radical, Reducing power

## Abstract

The aim of the present study was to evaluate the effect of troxerutin (TXN) on Nickel (Ni) toxicity by using rats and *in-vitro *model. Ni toxicity induced in male albino wistar rats (20 mg/kg body weight (b.w) was administered orally for 20 days). TXN was administered orally (100 mg/kg (b.w) for 20 days with administration of Ni. The toxic effect of Ni and the action of TXN was measure by determining the lipid peroxidation markers and antioxidant levels in plasma and various *in-vi*tro antioxidant systems. TXN exhibited a significant (*p *< 0.05) antioxidant activity in Ni induced toxicity by reversing the changes observed in TBARS, HP, Vitamin C, E and GSH. The free radical scavenging properties of TXN at different concentrations (10-50ug/mL) were investigated with various *in-vitro *methods such as 2, 2’-diphenyl-1- picrylhydrazyl radical (DPPH), 2, 2’-azinobis (3-ethylbenzothiazoline-6-sulfonic acid) radical (ABTS•+), hydroxyl radical, superoxide anion scavenging activity and reducing power. Among the different concentrations, 50 μg/mL of TXN was more effective compared to other concentrations in all *in-vitro *assays. The above study conclude that TXN possesses potent *in-vivo *and *in-vitro *antioxidant activity with effective free radical scavenger for potential therapeutic value

## Introduction

Ni occurs naturally in the earth’s crust and is ubiquitous in air, water, soil and the biosphere. The average concentration of Ni in the earth’s crust is 0.008% (1). Food and cigarette smokers are the main sources of Ni exposure in the general public approximately 0.04-0.58 μg Ni is released with the mainstream smoke of one cigarette. Smoking forty cigarettes a day may therefore lead to inhalation of 2-23 μg Ni. Nickel carbonyls is the most toxic Ni compound following acute exposure. The symptoms of acute exposure to nickel carbonyl occur in two stages, immediate and delayed. The immediate toxic effects of Ni carbonyl exposure are respiratory tract irritation and neurological symptoms. Initial symptoms include dizziness, frontal headache, nausea, vomiting, irritability and upper airway irritation (2)

Ni exerts its toxicity by generating reactive oxygen species (ROS) during its metabolism which results in the oxidative damage and depresses the antioxidant defense system (3). Reactive oxygen species (ROS) are capable of oxidizing cellular proteins, nucleic acids and lipids. Lipid peroxidation is a free-radical mediated propagation of oxidative insult to polyunsaturated fatty acids involving several types of free radicals, the most common reactive oxygen spices (ROS) include superoxide anion (O_2_.), hydroxyl radical (OH), hydrogen Peroxide (H_2_O_2_) proxy radical radicals (ROO). The nitrogen derived free radicals are nitric oxide (NO) and peroxynitriteanion (ONOO) (4). ROS have been implicated in over a hundreds of diseases states which range from arthritis and connective tissue disorders to carcinogenesis, aging, physical injury, infection and cardio vascular malfunction (5). An antioxidant is a molecule capable of slowing or preventing the oxidation of other molecules. In a biological system they may protect cells from damage caused by unstable molecules known as free radicals. Antioxidants terminate these chain reactions by removing free radical intermediates, and inhibit other oxidation reactions by oxidizing themselves. As a result, Antioxidants have been reported to prevent oxidative damage by free radical and ROS; any may prevent the occurrence of disease, cancer and aging. It can interfere with the oxidation process by reacting with free radicals, chelating, catalytic metals, and also by acting as oxygen scavengers (6).Most of the antioxidants commercially in use (e.g., butylated hydroxytoluene (BHT) and butyrate hydroxyanisole (BHA)) are synthetic and some of them have been suspected of causing or promoting negative health effects; therefore, some restrictions are placed on their applications and there is a trend to substitute them with naturally occurring antioxidants (7). So, attention has been drawn to the health promoting activity of plant foods and its active components. 

Flavonoids are a group of polyphenolic compounds diverse in chemical structure and characteristics. They are widely distributed in foods of plant origin such as vegetables, fruits, tea and wine (8). The antioxidant properties of various plants have been reported by different studies (9). The phenolic compounds in plants are found to be effective antioxidants due to their redox properties; they act as reducing agents (free radical terminators), hydrogen donors, singlet oxygen quenchers and metal chelators (10).

**Figure 1:**
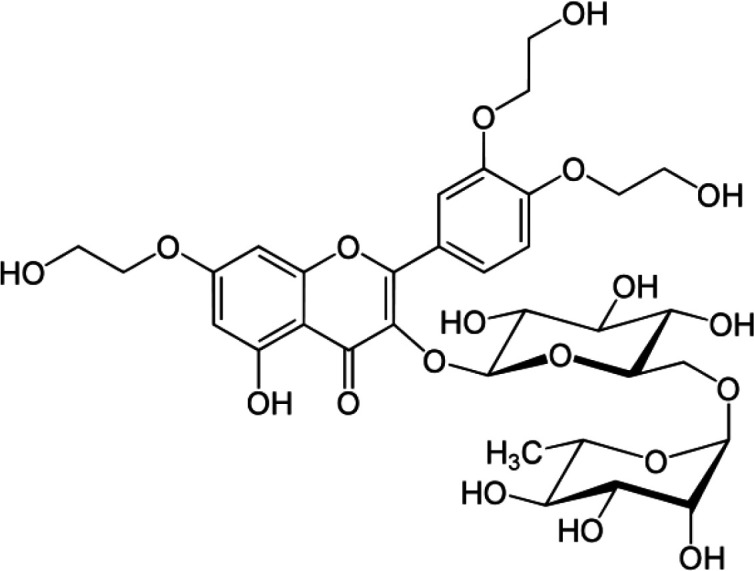
Structure of Troxerutin

Figure1.TXN, a trihydroxyethylated derivative of the natural bioflavonoidrutin, is present in tea, coffee, cereal grains and a variety of fruits and vegetables (11). Many reports have demonstrated that TXN possesses a variety of biological activities, such as anti-oxidative, anti-inflammatory, anti-neoplastic, ntierythrocytic, anti-thrombotic, anti-fibrinolytic, and anti-c-radiation induced DNA damage (12).The objective of the present study was to investigate the antioxidant activity of the TXN using different *in-vivo *and *in-vitro *models.

## Experimental

*Chemicals*

TXN, Ni sulfate, reduced glutathione (GSH), 2,2’-dipyridyl, 2,4-dinitro phenylhydrazine (DNPH), 5,5’-dithiobis-2-nitrobenzoic acid (DTNB), 2,2’-diphenyl-1-picrylhydrazyl radical (DPPH), 2,2’-azinobis (3-ethylbenzothiazoline-6-sulfonic acid) radical (ABTS) and butyrate hydroxytoulene were obtained from Sigma Chemical Co. (St. Louis, MO, USA). All other chemicals were obtained from S.D. Fine chemicals Mumbai, India and were of analytical grade.

*Animals*

Adult male albino rats of Wister strain (180 – 200 g) were used for the experiment. The animals were housed in polypropylene cages and maintained in 12-h light/12-h dark cycle, 50% humidity and 25 ± 2 °C. The animals had free access to standard pellet diet (M/S. Pranav Agro Industries Ltd., Bangalore, India) and water *ad libitum. *This study was approved (Vide.No.902, 2012) by Institutional Animal Ethics Committee of Annamalai University and the study conducted in accordance with the “Guide for the Care and Use of Laboratory Animals”.

*Experimental design*

The animals were randomly divided into four groups of six rats in each group.

**Group 1:** Control rats treated with isotonic saline (i.p.) for 20 days.

**Group 2: **Normal rats received TXN (100 mg/kg b.w/day) (35, 36) dissolved in water using Intragastric tube for 20 days.

**Group3**: Rats received Ni as Ni sulfate (20mg/kg b.wi.p). in isotonic saline For 20 days.

**Group4**: Rats received Ni (20mg/kg b.w.i.p) with oral administration of TXN100 mg/kg body weight) for 20 days. 

At the end of experimental period, animals in different groups were sacrificed by cervical decapitation under ketamine hydrochloride (30 mg/kg b.w) anesthesia. Blood was collected in a tube, heparinised for plasma. Plasma separated by centrifugation and used for various biochemical estimations.

*Biochemical assays*

*Estimation of lipid peroxidation*

Lipid peroxidation byproducts of TBARS and HP in plasma (0.5 mL) were estimated colorimetrically using the methods of Fraga* et al,* Jiang *et al *(13, 14) respectively. In brief, 0.1 mL of plasma was treated with 2 mL of TBA- TCA- HCl reagent (0.37% TBA, 0.25M HCl and 15% TCA, 1:1:1 ratio), placed for 15 min in a water bath and then cooled and centrifuged at 3500 ×g for 10 min at room temperature, the absorbance of clear supernatant was measured at 535 nm against a reference blank. Plasma (0.5 mL) was treated with 0.9ml of Fox reagent (88 mg of butylatedhydroxy toluene (BHT), 7.6mg of xylenol orange and 0.8mg of ammonium iron sulphate were added to 90mL of methanol and 10ml of 250 mM sulphuric acid and incubated at 37 °C for 30 min. Then the absorbance was read at 560nm.

*Determination of plasma non-enzymatic antioxidants*

Ascorbic acid (vitamin C) concentration was measured by Omaye *et al *(15) to 0.5 mL of plasma, 1.5 mL of 6% TCA was added and centrifuged (3500 ×g, 20 min). To 0.5 mL of supernatant, 0.5 mL of DNPH reagent (2% DNPH and 4% thiourea in 9N sulfuric acid) was added and incubated for 3 h at room temperature. After incubation, 2.5 mL of 85% sulfuric acid was added and color developed was read at 530 nm after 30 min. Vitamin E was estimated by the method of Desa (16). Vitamin E was extracted from plasma by addition of 1.6 mL ethanol and 2.0 mL petroleum ether to 0.5 mL plasma and centrifuged. The supernatant was separated and evaporated on air. To the residue, 0.2 mL of 0.2% 2,2-dipyridyl, 0.2 mL of 0.5% ferric chloride was added and kept in dark for 5 min. An intense red colored layer obtained on addition of 4 mL butanol was read at 520 nm. Reduced glutathione (GSH) was determined by the method of Ellman (17) Supernatant (1 mL) was treated with 0.5 mL of Ellman’s reagent (19.8 mg of 5, 5-dithiobisnitro benzoic acid in 100 mL of 0.1% sodium citrate) and 3.0 mL of phosphate buffer (0.2 M, pH 8.0) was added and the absorbance was read at 412 nm in spectrophotometer.

*Estimation of tissue protein*

 BSA (bovine serum albumin) was diluted serially to concentrations ranging from 5 to100 µg/ml. Each standard solution and sample were mixed with 10% TCA and immersed in boiling water for 15 min. After cooling at room temperature and centrifugation at 3700 × g for 20 min, the precipitate was resuspended by adding 5% TCA solution followed by centrifugation at 3700 × g for 20 min, and the supernatant was then removed. The precipitate was resuspended by addition of alkaline copper solution. The diluted phenol reagent was then added, and the precipitate solution was incubated for 30 min at 37 °C. For samples, an additional centrifugation step at 2000 × g for 5 min was included. The supernatant was then collected, and the absorbance was measured at 750 nm.

*Free radical scavenging activity*

The ability to scavenging the free radical, DPPH was measured as a decrease in absorbance at 517 nm by the method of Mensor *et al *(19). To a methanolic solution of DPPH (90.25 mmol), an equal volume of TXN (10-50 μg) dissolved in distilled water was added and made up to 1.0 mL with methanolic DPPH. An equal amount of methanol was added to the control. After 20 min, the absorbance was recorded at 517 nm in a UV-visible Spectrophotometer (Systronics).

*Total antioxidant activity assay*

Total antioxidant potential of TXN was determined by the ABTS assay, as described by Miller *et al*. (1996). The reaction mixture contained ABTS (0.002 M), TXN (10-50 μmol) and buffer in a total volume of 3.5 mL. The absorbance was measured at 734 nm in a UV- visible Spectrophotometer. 

*Superoxide anion scavenging activity*

Superoxide anion scavenging activity of TXN was determined by the method of Nishmiki *et al *(20) with modification. 1 mL of NBT (100 μmol of NBT in 100 mM phosphate buffer, pH 7.4), 1mL of NADH solution (14.68 μmol of NADH in 100 mmol phosphate buffer, pH 7.4) and varying concentration of TXN (10-50 μg) were mixed well. The reaction was started by the addition of 100 μmol of PMS (60 μmol/100 mmol of phosphate buffer pH 7.4). The reaction mixture was incubated at 30 ºC for 15 min. The absorbance was measured at 560 nm in a spectrophotometer. Incubation without TXN was used as blank. Decreased absorbance of the reaction mixture indicated increased superoxide anion scavenging activity.

*Hydroxyl radical scavenging assay*

The hydroxyl radical scavenging activity was determined by the method of Halliwell *et al *(21). The following reagents were added in the order stated below. The incubation mixture in a total volume of 1 mL contained 0.4 mL of 100 mmol of potassium dihydrogen phosphate-KOH buffer, varying volumes of TXN (10-50 μg/mL), 0.2 mL of 500 mmol of ferric chloride, 0.1 mL of 1 mmol of ascorbic acid, 0.1mL of 10 mmol of H_2_O_2_ and 0.2 mL of 2-deoxy ribose. The contents were mixed thoroughly and incubated at room temperature for 60 min. Then 1 mL of 1% TBA (1 gm in 100 mL of 0.05 N NaOH) and 1 mL of 28% TCA were added. All the tubes were kept in a boiling water bath for 30 min. The absorbance was read in a spectrophotometer at 532 nm with reagent blank containing distilled water in a place of TXN. The percentage scavenging activity was determined. Decreased absorbance of the reaction mixture indicated increased hydroxyl radical scavenging activity.

*Reducing power*

The reducing power was determined according to the method of Oyaizu (22). Different concentrations of TXN (10-50 μg/mL) were prepared in methanol mixed with phosphate buffer (2.5 mL, 0.2 M, pH 6.6) and potassium ferricyanide [K3[Fe (CN)_6_] (2.5 mL, 1%). The mixture was incubated at 50 °C for 20 min and 2.5 mL of TCA (10%) was added to the mixture, which was then centrifuged at 3000 rpm for 10 min. The upper layer of the solution (2.5 mL) was mixed with distilled water (2.5 mL) and FeCl_3_ (0.5 mL, 0.1%). The absorbance was measured at 700 nm. Increased absorbance of the reaction mixture indicated increased reducing power. Ascorbic acid was used as a standard.

*Statistical Analysis*

The data for various biochemical parameters were analyzed using analysis of variance (ANOVA) and the group means were compared by Duncan’s Multiple Range Test (DMRT). Values were considered statistically significant when *p *< 0.05.

## Results

*Effect of plasma lipid per oxidation*

Table.1shows the changes in vitamin C, vitamin E and GSH in plasma of normal and control rats. Vitamin C, vitamin E and GSH levels were significantly lower in Ni intoxicated rats than in control rats. In contrast Ni intoxicated rats treated with TXN led to significant increase in the plasma antioxidant levels of vitamin C, vitamin E and GSH.

Values are expressed in mean ± SD for n = 6 rats in each group. The levels of vitamin C, vitamin E and GSH in plasma are expressed as mg/dL. ^a-c^In each row, means with different superscript letter differ significantly at *p *< 0.05 (DMRT). 

Table.2 shows the levels of TBARS, and HP in plasma of control and experimental rats. Toxicity rats had elevated levels of TBARS, and HP in the plasma, when compared with normal and control rats. Rats treated with TXN significantly decreased the lipid per oxidation markers in toxicity in rats.

Values are expressed in mean ± SD for n = 6rats in each group. The levels of TBARS in plasma is expressed as moles/dL and hydroperoxides in plasma is expressed mmoles/dL.^ a-c^In each row, means with different superscript letter differ significantly at *p *< 0.05 (DMRT).

*In-vitro antioxidant activity*

Several concentrations ranging from 10-50 μg/mL of the TXN was tested for the antioxidant activity in different *in-vitro *models. It was observed that free radicals were scavenged by the test compounds in a concentration dependent manner in all the models.

Table.3 shows the free radical scavenging action of TXN on *in-vitro* study. TXN scavenges DPPH radical in a dose dependent manner (10-50 μg/mL). The DPPH radical scavenging activity was detected and compared with ascorbic acid. However, the highest percentage (37.9 %) scavenging activity of TXN was observed at 50 μg.

Inhibition of the ABTS radical in dose-dependent (10-50 μg/mL) was showed in table 4. The percentage scavenging activity of TXN increases with increase in concentration. However, the highest percentage (31.7%) scavenging activity was observed at 50 μg and compared with butyratehydroxytoulene.

Table.5 shows the scavenging effects of TXN on superoxide radical. TXN scavenges the above mentioned radicals *in-vitro *in a dose-dependent manner. The percentage scavenging activity of TXN increases with increasing concentration. The highest percentage (32.8%) of scavenging activity was observed at 50 μg/mL and compared with ascorbic acid.

Table.6 shows the scavenging effects of TXN on hydroxyl radical. TXN scavenges the abovementioned radicals *in-vitro *in a dose-dependent manner. The percentage scavenging activity of TXN increases with increasing concentration. The highest percentage (45.7%) scavenging activity was observed at 50 μg/mL and compared with ascorbic acid.

Table.7 shows the reducing power of TXN. The bars represent TXN and a positive control ascorbic acid. The reducing power of TXN and the reference compound, ascorbic acid were studied. Increased absorbance with the increased. Concentrations of the reaction mixture indicated the increased reducing power. However, the highest (0.034) scavenging activity was observed at 50 μg/mL.

## Discussion

The production of ROS is involved in the molecular mechanism of Ni toxicity and carcinogenicity (23). The role of free radicals and active oxygen in the pathogenesis of certain human diseases, including cancer, aging, and arteriosclerosis, is becoming increasingly recognized Georgakilas *et al *(24). In our present study, increase in the levels of TBARS and lipid hydro peroxides shows the involvement of enhanced lipid peroxidation, which may be due to increased free radical generation associated with decreased cellular levels of antioxidants in Ni induced toxicity. Treatment with TXN protects the cells through inhibition of lipid per oxidation as evidenced from the decreased levels of plasma TBARS and lipid hydroperoxides with the increased cellular levels of antioxidants. It clearly demonstrates the ability of TXN to directly interact with ROS that may initiate lipid per oxidation Ni toxic condition. It is well established that TXN act as a chain breaking antioxidant, it effectively quenches the free radicals thereby terminating the chain reaction of lipid per oxidation and minimizing its deleterious effects (25) May be due to the presence of hydroxyl group present in TXN.

Vitamin C is a primary antioxidant, water-soluble vitamin that can directly scavenge singlet oxygen, superoxide and hydroxyl radicals. Numerous reports have shown the positive effect of vitamin C as an antioxidant and scavenge of free radicals vitamin E is a well known physiological antioxidant and membrane stabilizer. It interrupts the chain reaction of LPO by reacting with lipid peroxy radicals, thus protecting the cell structures against damage (26). The decreased level of vitamin E observed in the toxic rats is compatible with the hypothesis that plasma vitamin E plays a protective role against increased per oxidation in toxicity.

GSH acts as a multifunctional intracellular non-enzymatic antioxidant and protects cells against several toxic oxygen-derived chemical species. It is considered to be an important scavenger of free radicals and a cofactor of several detoxifying enzymes against oxidative stress, e.g., glutathione peroxidase, glutathione-S-transferase (27). GSH is able to regenerate the most important antioxidants, vitamins C and E, back to their active forms (28). A constant supply of reduced GSH is necessary to repair the effects of spontaneous oxidation of sulfhydryl groups, which result in cell membrane damage (29). Several papers have reported that decreased levels of GSH after exposure to Ni. Treatment with TXN brought vitamin C, vitamin E and reduced glutathione to near normal levels which could be as a result of decreased membrane damage as evidenced by the antioxidant nature (23). Antioxidants exert their mode of action by suppressing the formation of reactive oxygen species by chelating trace elements. DPPH is widely used to evaluate the free radical scavenging effect of natural antioxidant. DPPH is a free radical generator at room temperature, which produces a violet solution in ethanol. DPPH shows a strong absorption at 517 nm. The assay is based on the measurement of the scavenging ability of antioxidants towards the radical DPPH. These radicals react with suitable reducing agents, the electrons become paired off and the solution loses color depending on the number of electrons taken up (30).

 Radical scavenging activity increased with increasing percentage of the free radical inhibition. Results indicated definite scavenging activity of the TXN towards DPPH radicals in comparison with ascorbic acid. The highest percentage scavenging effect of TXN on DPPH• at the concentration of 50 μg was 37.9%.

The ABTS assay is based on the inhibition of the absorbance of the radical cation ABTS+ which has a characteristic long wavelength absorption spectrum (31). The ABTS chemistry involves direct generation of ABTS+ radical mono cation with no involvement of any intermediary radical. It is an excellent tool for determining the antioxidant activity of hydrogen donating antioxidants and of chain breaking antioxidants. The total antioxidant activity was measured using the ABTS assay. In our study, inhibition of the ABTS^+^ radical shows dose-dependent manner. The highest percentage scavenging effect of TXN on ABTS^+^ at the concentration of 50 μg was 31.7%.

Superoxide dismutase is an antioxidant enzyme that neutralizes the free radicals in the cell; it dismutates superoxide anion (O_2_^-^) into H_2_O_2_ and protects the cells from damage by cleaning up O_2_^-^. The level of SOD activity represents the intracellular anti oxidation ability. Superoxide anion radical is one of the strongest reactive oxygen species among the free radicals that are generated. In the present study, superoxide radical reduces NBT to a blue colored formazan that is measured at 560 nm with antioxidants thus indicates the consumption of superoxide anions in the reaction mixture. In our study we have used different concentration of TXN (10-50 μg). The highest percentage scavenging effect of TXN on superoxide at the concentration of 50 μg was 32.8%.

 Hydroxyl radical scavenging activity was quantified by measuring the inhibition of the degradation of deoxyribose by the free radicals generated by the Fenton reaction. The oxygen derived hydroxyl radicals along with the added transition metal ion (Fe^2+^) causes the degradation of deoxyribose into malondialdehyde produces a pink chromogen with thiobarbituric acid. The scavenging activity of TXN was showed dose-dependent (10-50 μg) manner. However, the highest percentage scavenging activity was observed at 50 μg and the percentage was 45.7%. The antioxidant activity has been reported to have a direct, positive correlation with the reducing power (32).The reducing properties are generally associated with the presence of reductone, which have been shown to exert antioxidant action by breaking the free radical chain by donating a hydrogen atom (33). Reductones are also reported to react with certain precursors of peroxide, thus preventing peroxide formation (34). The reducing capacity of a compound may serve as a significant indicator of its potential antioxidant activity. Our data on the reducing power of the tested compound suggest that it is likely to contribute significantly towards the observed antioxidant effect. The reducing power of TXN increases with increasing amount of TXN was 0.034 at a dose of 50 μg showing that TXN can act as electron donors and can react with free radicals to convert them to more stable products and thereby terminate radical chain reactions.

According to data obtained, TXN significantly reduced lipid per oxidation (TBARS and LOOH) in plasma and increases the non-enzymatic antioxidant levels (vitamin C, E and GSH) mainly due to the antioxidant properties of TXN. This study has shown that the TXN at a dose of 50 μg exhibits highest free radical scavenging and antioxidant effects which has been proved by the methods of DPPH•, total antioxidant activity, superoxide anion scavenging activity, hydroxyl radical scavenging activity and reducing power during *in-vivo* and *in-vitro *model.

**Table 1:** Changes in the activities of vitamin- C, vitamin- E and reduced glutathione (GSH) in plasma of control and experimental rats

**Parameters**	**Control**	**Normal** **+Troxerutin(100 mg/kgb.w)**	**Normal** **+ Nickel** **(20 mg/kgb.w)**	**Nickel** ** (20 mg/kg** **b.w** **)** **+Troxerutin(100 mg/kgb.w)**
**Vitamin C **				
Plasma	2.88 ± 0.26^ a^	2.94 ± 0.27^ a^	1.97 ± 0.16^ b^	2.48 ± 0.22^c^
**Vitamin E**				
Plasma	2.35 ± 0.21^a^	2.38 ± 0.24^a^	1.65±0.14^b^	1.98 ± 0.17^ c^
**GSH **				
Plasma	18.77 ± 1.63^a^	19.25 ± 1.72^a^	13.91 ±1.06^b^	16.15 ± 1.44^c^

**Table 2:** Changes in the levels of TBARS and LHP in the plasma of normal control and experimental rats

**Parameters **	Control	Normal +Troxerutin (100 mg/kgb.w)	Normal + Nickel (20 mg/kgb.w)	Nickel (20 mg/kgb.w) +Troxerutin (100 mg/kgb.w)
TBARS				
Plasma	0.14 0.01^a^	0.13 0.01^a^	0.22 0.01^b^	0.17 0.01^c^
Lipid hydroperoxides				
Plasma	11.75 1.12^a^	11.30 1.09^a^	19.24 1.82^b^	15.21 1.42^c^

Values are expressed in mean SD for n = 6rats in each group. The levels of TBARS in plasma is expressed as moles/dL and hydroperoxides in plasma is expressed mmoles/dL.^ a-c^In each row, means with different superscript letter differ significantly at *p *< 0.05 (DMRT).

**Table 3:** DPPH-free radical scavenging action of TXN on *in-vitro *assay

**DPPH Concentration (μg/mL)**	**Ascorbic acid (%)**	**Troxerutin (**TXN) **(%)**
10	9.54	7.5
20	19.72	17.8
30	28.76	24.7
40	38.9	33.4
50	51.7	37.9

Values are expressed in mean ± SD of triplicate.

**Table 4:** ABTS- total antioxidant scavenging assay

**ABTS Concentration (μg/mL)**	**Ascorbic acid (%)**	**Troxerutin (%)**
10	7.99	6.4
20	15.42	13.2
30	23.92	19.9
40	29.36	25.8
50	36.92	31.7

Values are expressed in mean ± SD of triplicate.

**Table 5:** Superoxide radical scavenging assay

**Superoxide radical Concentration (μg/mL)**	**Ascorbic acid (%)**	**Troxerutin (%)**
10	8.76	6.15
20	15.4	12.4
30	26.4	19.6
40	33.6	25.4
50	41.5	32.8

Values are expressed in mean ± SD of triplicate.

**Table 6:** Hydroxyl radical scavenging assay

**Hydroxyl radicalConcentration (μg/mL)**	**Ascorbic acid (%)**	**Troxerutin (%)**
10	11.27	8.68
20	23.15	17.21
30	32.8	26.4
40	44.12	34.5
50	55.67	45.7

Values are expressed in mean ± SD of triplicate.

**Table 7:** Reducing power

**Reducing power** ** Concentration (μg/mL)**	**Ascorbic acid **	**Troxerutin**
	**Absorbance at 700 nm**	
10	0.01	0.007
20	0.018	0.013
30	0.028	0.023
40	0.039	0.027
50	0.052	0.034

Values are expressed in mean ± SD of triplicate.
